# Surveillance After a Previous Cutaneous Melanoma Diagnosis: A Scoping Review of Melanoma Follow-Up Guidelines

**DOI:** 10.1177/12034754231188434

**Published:** 2023-07-25

**Authors:** Leah Johnston, Samantha Starkey, Ilya Mukovozov, Lynne Robertson, Teresa Petrella, Raed Alhusayen

**Affiliations:** 1 70401 Cumming School of Medicine, University of Calgary, Calgary, Canada; 2 12358 Faculty of Medicine, University of British Columbia, Vancouver, BC, Canada.; 3 12358 Department of Dermatology and Skin Science, University of British Columbia, Vancouver, BC, Canada; 4 2129 Division of Dermatology, University of Calgary, Calgary, AB, Canada; 5 71545 Department of Medical Oncology, Odette Cancer Centre, Toronto, Canada; 6 71545 Sunnybrook Research Institute, Division of Dermatology, University of Toronto, Toronto, Canada

**Keywords:** melanoma, skin cancer, surveillance, clinical practice guidelines, follow-up, review

## Abstract

**Introduction:**

Cutaneous melanoma accounts for more than 70% of all skin cancer deaths. Follow-up surveillance is an integral part of melanoma patient care, to facilitate early detection of recurrences and subsequent primary melanomas. The purpose of this scoping review is to provide an overview of recently published melanoma surveillance guidelines from regional and national melanoma working groups.

**Methods:**

A systematic search for relevant studies in MEDLINE and Embase was conducted in September 2022 and was limited to publications from 2010 or later.

**Results:**

A total of 1047 articles were retrieved, and after abstract and full text review, 26 articles from 19 different organizations met inclusion criteria. Life-long annual skin surveillance with a physician was recommended by 53% (9/17) of guidelines. Routine laboratory investigations were recommended by 7/19 guidelines. Regional lymph node ultrasound was recommended by 9/16 guidelines, most often in stage IB or higher, and was optional in 7/16 for patients who met specific criteria. Surveillance with PET-CT or CT and MRI was recommended by 15 and 11 guidelines, respectively, most commonly in stage IIC or higher, with a variable frequency and total duration. Five out of 9 guidelines indicated a preference for skin surveillance to be completed with a dermatologist.

**Conclusion:**

Guidelines were highly variable for many aspects of melanoma surveillance, which may be partly attributed to regional differences in healthcare workforce distribution and availability of imaging technologies. Further high-level studies are recommended to provide more evidence on the most effective clinical and imaging follow-up surveillance protocols.

## Introduction

Cutaneous melanoma accounts for 5.2% of all new cancer diagnoses.^1^ More than 70% of all deaths from skin cancer can be attributed to melanoma, due to its high metastatic potential.^2^ In recent decades, there has been an increase in the incidence and prevalence rates of malignant melanoma in developed countries.^2,3^ As a result of both genetic and environmental risk factors, individuals with a previous history of melanoma are at risk for developing subsequent primary melanomas.^4,5^

Additionally, individuals with melanoma are at risk for developing recurrences after definitive management of the primary cutaneous tumor, which includes metastases to distant organs. Distant metastases have a significant adverse impact on melanoma survival rates.^6^ With the development of new therapies to treat metastatic melanoma, it is possible that survival rates for metastatic melanoma may improve, especially if metastases are detected at an early stage.^7^ Complete surgical resection of oligometastases has also demonstrated a survival benefit but is typically limited to patients with isolated disease.^8^

To help facilitate early detection of recurrences and subsequent primary melanomas, individuals with cutaneous melanoma are recommended to undergo clinical follow-up and, in some cases, routine surveillance imaging, in the years following the initial melanoma diagnosis. However, limited high-quality studies have been conducted to investigate the use of follow-up surveillance in melanoma patients. Additionally, the delivery of follow-up care may be impacted by regional availability of healthcare resources, including specialist physician care and surveillance imaging modalities. The purpose of this review is to provide an overview of recent international guidelines on follow-up care of melanoma patients and an analysis of the supporting evidence for surveillance recommendations.

## Materials and Methods

### Literature Search

A database search for relevant studies in MEDLINE and Embase was conducted on September 10, 2022 using the following MeSH keywords: (‘melanoma’) AND (‘follow-up’ OR ‘surveillance’) AND (‘guidelines’ OR ‘recommendations’). A manual search of reference lists of included studies was performed to ensure all relevant studies were captured. To accurately reflect recent research advancements and current clinical practice, the literature search was limited to articles that were published from January 1, 2010 to September 10, 2022. Title, abstract, and full-text screening was completed in duplicate by two reviewers (L.J. and S.S.).

### Eligibility Criteria

National and international guidelines that contained recommendations on melanoma follow-up surveillance were eligible for inclusion. Guidelines were required to contain recommendations on at least one of either (i) clinical follow-up or (ii) surveillance investigations. If multiple versions of a guideline publication were published by a particular melanoma expert group, only the most recent publication was included in the review.

Articles were excluded if the primary focus was not cutaneous melanoma. Additionally, publications which focused on staging or re-staging of symptomatic recurrent disease or monitoring of response to treatment were also excluded, as the focus of this review is on routine follow-up surveillance.

### Data Extraction

Data extraction was completed by two reviewers (L.J. and S.S.). The Preferred Reporting Items for Systematic Reviews and Meta-Analyses Extension for Scoping Reviews checklist was used as a guide for development of this article.^9^

## Results

The MEDLINE and Embase database searches yielded 1047 articles (Supplemental Figure 1). Following title and abstract screening, a total of 87 publications underwent full text review and 18 met criteria for inclusion. An additional 8 citations from 3 different organizations (Cancer Council Australia, National Institute for Health and Care Excellence and the National Comprehensive Cancer Network) were identified after manual review of the citations in included articles.

### National Clinical Practice Guidelines

Nineteen different national clinical practice guidelines were referenced from the 26 articles that met the inclusion criteria (Supplemental Table 1). Countries that were represented by the published guidelines included Australia, Brazil, Canada, Croatia, France, Germany, Italy, New Zealand, Poland, Spain, Switzerland, the United Kingdom, and the United States. Multinational European guidelines were also included.^10-35^

### Patient Education on Sun Safety

Patient education on sun safety was discussed and recommended by 14 clinical practice guidelines (Supplemental Table 2). Specific sun safety recommendations included variations of the following: (i) minimizing sun exposure during peak daylight hours, (ii) wearing sun-protective clothing, sunglasses, and a wide-brimmed hat, and (iii) applying broad-spectrum ultraviolet (UV) sunscreen regularly to sun-exposed skin when outdoors.

### Self-Skin Examinations

Self-skin examination was discussed by 17 guidelines (Supplemental Table 2). All 17 guidelines recommended that patients routinely complete self-exams. Individual recommendations varied amongst guidelines, but the following were frequently occurring instructions for patients: examination of the entire skin surface, examination of the surgical scar, and/or palpation of the regional lymph node basins. In the five guidelines that recommended a specific frequency of self-skin exams, the recommended frequency varied from once monthly to annually.^17,25,27,29,32^

### American Joint Committee on Cancer Staging and Definition of High-Risk Melanoma Group

The American Joint Committee on Cancer (AJCC) staging system for melanoma was used in all 19 clinical practice guideline publications. The seventh edition of the AJCC melanoma staging guidelines was published in 2009 and was used in 10 guidelines.^36^ The eighth edition was published in 2017 and was incorporated into 9 of the guidelines (Supplemental Table 2).^37^

Most guidelines (18/19) provided a definition for the high-risk category of melanoma patients based on AJCC stage. The most frequent definition of a high-risk surveillance group began at stage IIB (8/19 guidelines), while 7 guidelines selected stage IIC and 3 guidelines chose stage III as the earliest stages included in high-risk surveillance schedules (Supplemental Table 2). The Brazilian Society of Dermatology guidelines only proposed recommendations for stage 0-II and did not provide surveillance guidelines for more advanced stages.

### Recommended Duration of Clinical Skin Surveillance

Seventeen guidelines provided recommendations for the total duration of skin surveillance with a healthcare provider (Supplemental Table 2). The recommendations for clinical surveillance can be stratified into two main categories: (i) life-long surveillance and (ii) fixed-duration surveillance. The most common recommendation was life-long surveillance at an annual frequency, which was recommended by 53% (9/17) of the guidelines. Fixed duration of clinical surveillance following a patient’s initial diagnosis or most recent change in AJCC staging was recommended by 47% (8/17) of the guidelines. Of these 8 guidelines, 4 recommended that routine clinical surveillance occur for a maximum time period of 10 years. The Canadian Melanoma Conference guidelines focused exclusively on surveillance in high-risk (stage IIB-IV) patients and recommended clinical surveillance for 5 years.^18^ Three guidelines recommended a stage-dependent, variable fixed-duration schedule. The Network of Catalan and Balearic Melanoma Centers recommended that follow-up occur for a duration of 3-5 years in melanoma in situ, while stage IB-IV melanoma patients should undergo follow-up for 10 years.^29^ Two guidelines from organizations in the United Kingdom, the National Institute for Health and Care Excellence and the British Association of Dermatology, recommended that patients with melanoma in situ may be discharged after post-surgical follow-up and do not require ongoing surveillance, while patients with advanced AJCC stages should be followed for up to 5 and 10 years, respectively.^32,33^

### Clinical Follow-Up Schedules

Recommendations for clinical follow-up schedules are provided in 17 of the 19 guidelines (Supplemental Table 3). New Zealand’s National Melanoma Working Group and the European Society of Medical Oncology guidelines were not included in the tables. The National Melanoma Working Group guidelines focus exclusively on surveillance imaging and did not discuss healthcare provider-led clinical examination and the European Society of Medical Oncology guidelines did not present a specific timeline for clinical follow-up.

In the 17 guidelines that contained clinical follow-up recommendations, all guidelines recommended a stage-specific approach to determining the frequency of follow-up (Supplemental Table 3). Each guideline stratified follow-up groups into low risk, intermediate risk and/or high-risk categories based on AJCC stage, with more frequent follow-up recommended in more advanced stages. Additionally, 16/17 guidelines recommended more frequent follow-up during the initial years of the total follow-up period. The period of more intensive surveillance occurred during the first 1-5 years after initial diagnosis, definitive management, or re-staging after disease progression. This was followed by a period of less frequent surveillance, then either stopping routine clinical surveillance completely or reducing the frequency to an annual, life-long schedule. The CMC guidelines were the only guidelines that recommended a fixed frequency of follow-up for the full duration of the surveillance period, with 6-monthly visits scheduled for the entire 5-year period of clinical surveillance.

### Recommendations for Which Specialists Should Conduct Follow-Up Surveillance Visits With Melanoma Patients

Nine guidelines included recommendations for the medical disciplines and experience of providers who should be involved in the clinical follow-up care of melanoma patients (Supplemental Table 3). Out of these 9 guidelines, 6 indicated a preference for one or two types of specialists, while 3 guidelines suggested multiple different disciplines that could oversee follow-up care. Five guidelines indicated that having clinical skin examinations with a dermatologist is preferrable and one guideline stated that clinical skin surveillance should be completed with either a dermatologist or a primary care physician. In the three guidelines that did not prefer a particular discipline to oversee follow-up, dermatologists, general practitioners, medical oncologists, and surgeons were listed as potential candidates.

Six guidelines discussed expertise in dermoscopy as an important skill for skin surveillance providers to offer to their melanoma patients (Supplemental Table 3). The Cancer Council Australia guidelines recommended that follow-up skin surveillance with a provider who has experience in skin examination would be preferrable.^10-16^ The European Dermatology Forum, European Association of Dermato-Oncology and European Organization for Research and Treatment of Cancer guidelines preferred follow-up with a dermatologist due to their focused clinical training on skin examination and dermoscopy.^21^ However, as some European countries may have a limited number of practicing dermatologists, follow-up with a general practitioner that has completed additional training in dermoscopy is a suitable alternative.

### Laboratory and Radiographic Surveillance Investigations

Specific recommendations on follow-up imaging are outlined in Supplemental Table 4. Investigations that were discussed by at least half of the included guidelines are summarized below. These included laboratory tests, regional lymph node ultrasound (US), chest x-ray (CXR), positron emission tomography with computed tomography (PET-CT), conventional CT with contrast, and brain magnetic resonance imaging (MRI).

### Regional Lymph Node Ultrasound

Regional lymph node US was discussed by 16 guidelines, recommended by 9 and listed as optional by 7 guidelines (Supplemental Table 4). Of the guidelines which indicated that lymph node US is optional, 6 guidelines stated that lymph node US should be performed in patients who either did not undergo a sentinel lymph node biopsy (SLNB) as recommended or in individuals with a known positive SLNB who did not undergo a complete lymph node dissection. Fifteen guidelines recommended a specific stage to start regional lymph node US. Eight guidelines suggested stage IB as the earliest stage to perform regional lymph node US. The remaining 7 guidelines recommended starting lymph node US at stage IA (n=1), IIA (n=1), IIB (n=1), IIC (n=2), and III (n=2).

### Chest X-Ray

CXR was recommended by 2 publications, not recommended by 5, and listed as an optional investigation in 3. In guidelines that did not discuss CXR or recommended against its use in follow-up care, explanations included the use of full body PET-CT or chest CT as preferred surveillance investigations for lung metastases or a general lack of robust evidence to support the use of surveillance imaging.

### PET-CT And/or CT

PET-CT or CT for follow-up surveillance was recommended by 15 of the 19 clinical practice guidelines (Supplemental Table 4). The National Institute for Health and Care Excellence guidelines did not recommend specific imaging modalities but stated that imaging may occur at stage IIC or higher. Of the 17 guidelines that recommended imaging at a particular stage, 7 recommended IIC as the earliest AJCC stage to incorporate imaging into follow-up surveillance. Six guidelines recommended starting surveillance at stage IIB, one recommended stage II and two recommended stage III. The Cancer Council Australia guidelines suggested that follow-up imaging may be useful in patients with stage IIC or higher disease, but the potential for change in management based on findings should be taken into consideration when determining whether to incorporate PET-CT or CT imaging into a surveillance plan.

Eight guidelines indicated a preference for PET-CT over conventional CT of the chest, abdomen, and pelvis (CT-CAP), due a higher sensitivity and specificity of PET-CT for detection of distant metastases. CT was preferred by 6 publications and one publication suggested that either PET-CT or CT may be performed, without a statement indicating a preference for either test. Limited regional accessibility of PET-CT and higher cost compared to conventional CT were listed as reasons for recommending CT over PET.

The total duration of routine PET-CT or CT imaging ranged from 3 to 5 years in 14 guidelines (Supplemental Table 4). The frequency of imaging was variable, ranging from every 3 months to every 12 months. Eight guidelines recommended a fixed frequency of imaging throughout the full surveillance period, while 6 guidelines recommended more frequent imaging in the first few years of surveillance, followed by a period of reduced frequency of surveillance until the end of the imaging surveillance period. Four guidelines recommended stage-adjusted frequencies, with less frequent imaging in earlier stages and more frequent imaging in the most advanced stages.

### Brain MRI

Eleven guidelines recommended the use of brain MRI for surveillance of cerebral metastases (Supplemental Table 4). Two guidelines listed brain MRI as an optional investigation and six guidelines did not discuss brain MRI. Six guidelines suggested that brain MRI should be performed in patients with stage IIC or higher disease, while 5 guidelines suggested starting MRI surveillance at stage IIB. The recommended total duration of MRI surveillance ranged from 3 to 5 years. The frequency of MRI ranged from 3-monthly to 12-monthly intervals. Most guidelines recommended a fixed frequency of imaging throughout the surveillance period. Two guidelines recommended a higher initial frequency of imaging, followed by a period of reduced frequency surveillance prior to the end of the imaging surveillance period.

### Laboratory Investigations

Laboratory investigations were recommended for follow-up surveillance by 7/19 publications and were recommended against by 6 guidelines (Supplemental Table 5). Three publications indicated that laboratory investigations were optional and 3 guidelines did not discuss the use of laboratory investigations in follow-up care. In the guidelines that recommended specific laboratory investigations or listed them as optional, S100B and lactate dehydrogenase (LDH) were listed as potential surveillance tests.

## Discussion

This study summarizes national and international guidelines that were published by melanoma specialist organizations. Outcomes investigated included patient education, self-skin examination, total duration of skin surveillance, definition of high-risk melanoma patients, frequency of clinical surveillance visits with a physician, recommendations for surveillance investigations, and recommendations for specialty of physicians who oversee the care of patients with melanoma.

The recommendations with the highest agreement amongst publications were the guidelines on patient education on sun safety and self-skin examinations. As UV exposure is a known risk factor for melanoma, counselling patients on methods to minimize UV-mediated damage to the skin is essential to prevent additional melanomas. One study found that patients who reported infrequently or never using sunscreen after a first primary melanoma diagnosis had a 2-fold increased risk of developing subsequent primary melanomas compared to individuals who frequently used sunscreen.^38^ Additionally, a recent literature review of the evidence on sun safety recommendations found that there is strong evidence to support that the risk of both melanoma and non-melanoma skin cancers may be significantly reduced by daily sunscreen application.^39^ Patients with melanoma should be advised to minimize sun exposure during peak daylight hours, wear sun-protective clothing, sunglasses and a wide-brimmed hat, and apply broad-spectrum UV sunscreen regularly to exposed skin when outdoors.^35,39^

Educating patients on both the importance of self-skin examination and how to perform self-examinations was emphasized by most clinical practice guidelines. Previous studies on self-skin examinations have found that regular self-skin examinations may lead to earlier detection of melanoma and reduced mortality.^40,41^ Some studies have found that the majority of melanoma recurrences were initially detected by patients or their family members, compared to detection by a healthcare provider or with surveillance imaging, especially if the recurrences were locoregional.^42-44^ This demonstrates that regular self-examinations are an essential component of a melanoma surveillance plan. Monthly self-skin and lymph node examinations have the strongest support from the literature, although randomized controlled trials (RCTs) have not been conducted to investigate the optimal frequency of self-examinations in patients with melanoma.^45^

Recommendations for clinical follow-up were highly variable. Recent studies have found that the peak incidence rates of melanoma recurrences and subsequent primary melanomas occur during the first 2-3 years after a melanoma diagnosis.^5,42,44,46-61^ Overall, less than 5% of melanoma recurrences occur beyond 5 years after definitive management.^15^ However, several studies have suggested that there is an elevated risk of subsequent primary melanomas that persists beyond 10 years after the initial melanoma diagnosis.^46,60,62-65^ Therefore, combining time-limited clinical follow up with life-long self-skin examination could be a reasonable approach.

The frequency of clinical surveillance varied amongst guidelines. All guidelines recommended a stage-specific approach to follow-up schedules, with follow-up frequencies ranging from every 3 months to annually. The 2016 melanoma follow-up (MELFO) trial in Australia investigated follow-up frequencies in stage IB-IIC melanoma patients and found that there were no differences in detection of recurrences with a reduced frequency, stage-specific follow-up schedule compared to higher frequency surveillance schedules.^66^ In stage II melanoma, the reduced-frequency MELFO follow-up schedule recommends a higher intensity surveillance period consisting of 2-3 visits per year for the first 2 years, followed by 1-2 visits per year in year 3 and then annual visits in years 4-5.^66^

The guidelines did not indicate a strong preference for the specialties of healthcare providers who should oversee follow-up care. However, routine surveillance with a provider who has dermoscopy training and experience with conducting skin examinations was consistently recommended. A previous study that compared general practitioner (GP)-based follow-up in Scotland to specialist follow-up at a tertiary care center found that with brief additional training in skin surveillance, GP-led follow-up had similar health outcomes and high patient satisfaction ratings.^67^ In the context of the small number of dermatologists in Canada, GP-led follow-up, especially in low-risk melanomas, could be a sustainable approach.

Lymph node ultrasonography is the preferred surveillance test for regional lymph nodes. Previous studies have found that lymph node US is superior to clinical examination for detection of lymph node metastases and it has a sensitivity of 96% and a specificity of 99%.^68,69^ In this review, 9/16 guidelines recommended routine lymph node US and 7/16 studies listed US as an optional imaging modality or suggested routine use only in individuals who did not undergo SLNB or complete lymph node dissection after a positive SLNB. Evidence regarding the utility of regional lymph node ultrasonography in routine surveillance of early-stage disease is inconclusive. A cohort study of 1149 stage IB and IIA melanoma patients found that there was no survival benefit of routine ultrasonography.^70^ However, the risk of lymph node metastases is higher in patients with more advanced disease and routine ultrasonography may be beneficial in patients with stage IIB or higher disease. A 2011 study by Krüger et al. found that in a cohort of 433 patients who were predominantly in stages II-IV, 67% (22/33) of lymph node recurrences were detected by ultrasonography in the absence of clinical suspicion.^71^ Bartlett et al. found that regional lymph node ultrasonography every 3-4 months in SLNB-positive patients was useful in identifying recurrences at a surgically resectable stage in the 13.0% of patients who experienced recurrence.^72^

The use of PET-CT, conventional CT-CAP and/or MRI was discussed and recommended by most guidelines. Imaging surveillance was not recommended in patients with stage 0-IIA melanoma, as detection rates of distant recurrences by imaging tend to be lower than the false positive rates.^73^ Most guidelines indicated that surveillance imaging should start at either stage IIB or IIC. Patients with stage 0-IIA melanoma are more likely to develop locoregional metastases than distant metastases, which are more frequently detected by self-exam or physician-led physical examination.^74^ Conversely, multiple studies have found that stage IIB and IIC patients have much higher rates of distant recurrences and detection of these recurrences by imaging. Approximately half of all patients with stage IIB, IIC and III melanoma had recurrences detected by surveillance PET-CT or CT in previous studies.^48,73^ A 2011 systematic review found that the sensitivity and specificity of PET-CT (86% and 91%) was significantly higher than conventional contrast-enhanced CT (63% and 78%) of the chest, abdomen and pelvis and PET alone (82% and 83%).^69^ However, CT-CAP may still be an acceptable choice in geographic regions with limited access to PET-CT and this was cited as a reason for recommending CT-CAP over PET-CT by some guidelines. CXR was shown to be inferior for detection of pulmonary metastases compared to CT in a previous prospective cohort study and was not recommended by most clinical practice guidelines.^48^ For screening to rule out potential brain metastases, MRI is the preferred imaging modality, as it has a higher resolution and can detect smaller metastases than CT and PET-CT.^75^

One potential concern with the use of routine surveillance imaging is the high rate of false positive test results. The false positive rate for PET-CT has been estimated to be between 7-14% and for conventional CT, the rate is up to 20%.^33,74,76-80^ One study estimated that 46% of patients had at least one false-positive result over a 5-year period with PET-CT or CT imaging every 6-12 months.^81^ A significant portion of patients with false positive imaging results subsequently undergo unnecessary diagnostic imaging and invasive surgical procedures.^76,79-81^ The potential benefits of early detection of recurrences must be balanced with the potential harms of unnecessary interventions and additional stress that may result from imaging surveillance. Additionally, high-quality evidence that supports a survival benefit from routine surveillance imaging is lacking in the current literature.^74,76^ However, with new emerging systemic therapies for metastatic melanoma, the survival benefits of surveillance imaging may become more evident.^74,76^

Guidelines on the use of routine laboratory testing differed substantially, with 63% (10/16) of guidelines recommending against routine use of laboratory tests for surveillance. Of the guidelines that recommended specific laboratory tests or listed optional tests, S100B and LDH were most frequently included. Serum S100B is secreted by human melanoma cells and elevated levels may indicate disease progression, presence of metastases and reduced survival odds.^82,83^ However, S100B is also expressed by many different cell lineages and elevations have been associated with numerous other pathological states, including myocardial infarction, liver cirrhosis and chronic kidney disease.^82^ In one study that followed 1113 stage IB-IV melanoma patients over 2 years, 16% had false-negative and 12% had false-positive S100B results.^82^ The sensitivity and specificity of S100B were 55% and 88%, respectively.^82^ LDH is also used as a biomarker in melanoma staging to predict prognosis, but a previous study found that LDH had a low sensitivity for detecting new metastases during follow-up surveillance.^83^ Both S100B and LDH are unable to detect locoregional metastases and may not be useful in patients with early-stage disease.

The role of serum laboratory testing in melanoma follow-up surveillance must be considered in the context of other surveillance modalities. Laboratory tests are often not the first sign of metastasis and more definitive radiologic imaging is needed to definitively determine if distant metastases are present.^48,83-85^ In a 2016 prospective cohort study that followed stage IIB, IIC and III melanoma patients for a median of 2.5 years, only 2.5% of patients had increased serum S100B or melanoma inhibitory activity protein as the first sign of metastasis.^48^

Overall, the quality of supporting evidence for many follow-up surveillance recommendations was limited, especially for surveillance imaging, due to a lack of RCTs that have been conducted on follow-up surveillance schedules. This was a major limitation that likely led to the significant differences in recommendations that were observed amongst the included studies in this review. Further study in RCTs is needed to investigate the utility of surveillance imaging in the follow-up care of melanoma patients. Another limitation in this review was the diversity of approaches within the 19 included guidelines with respect to evaluating the supporting evidence and strength of their recommendations. Six guidelines did not discuss the use of a particular evidence appraisal scheme and did not provide levels of evidence or evidence grading. Of the 13 guidelines that provided an assessment of evidence, different evidence appraisal guidelines were used, making it challenging to provide a quantitative comparison between guidelines. Additionally, most guidelines took both the available supporting evidence and the clinical experience of expert panelists into consideration to develop guidelines with real-world applicability to a particular geographic region. National healthcare systems may differ in terms of access to specialist care and imaging studies and some recommendations from national guidelines may not be applicable to the healthcare systems of other countries.

In conclusion, this scoping review of global melanoma follow-up surveillance guidelines has demonstrated that there are significant differences in recommendations from different working groups and geographic regions. Further high-level studies are needed to provide more evidence on the most effective clinical and imaging follow-up surveillance protocols. In addition, careful consideration of disease prevalence, healthcare structure, workforce composition, and resources should be applied when developing guidelines for a specific jurisdiction or geographic location.

## Supplemental Material

Supplementary Material 1 - Supplemental material for Surveillance After a Previous Cutaneous Melanoma Diagnosis: A Scoping Review of Melanoma Follow-Up Guidelines Click here for additional data file. Supplemental material, Supplementary Material 1, for Surveillance After a Previous Cutaneous Melanoma Diagnosis: A Scoping Review of Melanoma Follow-Up Guidelines by Leah Johnston, Samantha Starkey, Ilya Mukovozov, Lynne Robertson, Teresa Petrella and Raed Alhusayen in Journal of Cutaneous Medicine and Surgery

Figure S1 - Supplemental material for Surveillance After a Previous Cutaneous Melanoma Diagnosis: A Scoping Review of Melanoma Follow-Up Guidelines Click here for additional data file. Supplemental material, Figure S1, for Surveillance After a Previous Cutaneous Melanoma Diagnosis: A Scoping Review of Melanoma Follow-Up Guidelines by Leah Johnston, Samantha Starkey, Ilya Mukovozov, Lynne Robertson, Teresa Petrella and Raed Alhusayen in Journal of Cutaneous Medicine and Surgery
