# Supplementary material for: Surveillance After a Previous Cutaneous Melanoma Diagnosis: A Scoping Review of Melanoma Follow-Up Guidelines
Source: J Cutan Med Surg. 2023 Jul 25;27(5):516–25. doi: 10.1177/12034754231188434 (PMC10617001; doi:10.1177/12034754231188434)

**Surveillance After a Previous Cutaneous Melanoma Diagnosis:**

**A Scoping Review of Melanoma Follow-Up Guidelines**

**Supplementary Table 1.** Guideline Development Methodology used in Clinical Practice Guidelines.

| **Geographic Region/Guideline** | **Publication Year** | **Literature Review Methods** | **Specialities in Expert Panel** | **Number of Panel Members** | **Evidence Appraisal (Levels of Evidence and/or Evidence Grading)** |
| --- | --- | --- | --- | --- | --- |
| Australia (CCA)^11-17^ | 2018-2020 | SR | D, GS, MO, PC | 15 | Y |
| Brazil (SBD)^18^ | 2016 | LR | D | 11 | Y |
| Canada (CMC)^19^ | 2021 | LR | AH, D, MO, P, R, RO, SO | 26 | N |
| Croatia (CSMO)^20,21^ | 2016-2017 | LR | MO | 40 | Y |
| Europe (EDF/EADO/EORTC)^22^ | 2022 | SR | D, MO, P, R, SO | 20 | Y |
| Europe (ESMO)^23^ | 2019 | LR | D, MO, SO | 5 | Y |
| France (SFD)^24^ | 2017 | SR | D, MO, P, R, RO, SO | 15 | Y |
| Germany (DDG/DeCOG)^25^ | 2013 | SR | D, MO, R, RO, SO | 65 | Y |
| Italy (IRCCS Reggio Emilia)^26^ | 2017 | SR | D | 6 | N |
| New Zealand (NMWG)^27^ | 2021 | LR | R, SO | 4 | N |
| Poland (MCMCC)^28^ | 2014 | LR | MO | 2 | N |
| Spain (FESEO)^29^ | 2022 | LR | D, MO, P, R | 8 | N |
| Spain (Network of Catalan and Balearic Melanoma Centers)^30^ | 2010 | LR | D | 25 | N |
| Spain (SEOM/GEM)^31^ | 2021 | LR | MO | 10 | Y |
| Switzerland (SAKK)^32^ | 2016 | LR | D, MO, P, RO, SO | 11 | Y |
| United Kingdom (BAD)^33^ | 2010 | LR | AH, D, MO, R, P, PC, SO | 38 | Y |
| United Kingdom (NICE)^34^ | 2015 | SR | AH, D, MO, P, PC, R, SO | 17 | Y |
| United States (AAD)^35^ | 2019 | SR | D, MO, P, SO | 16 | Y |
| United States (NCCN)^36^ | 2022 | LR | D, MO, P, RO, SO | 34 | Y |

AAD, American Academy of Dermatology; AH, allied health; BAD, British Association of Dermatologists; CCA, Cancer Council Australia; CMC, Canadian Melanoma Conference; CMSO, Croatian Society for Medical Oncology; D, dermatology; DDG/DeCOG, German Dermatological Society and the Dermatologic Cooperative Oncology Group; EADO, European Association of Dermato-Oncology; EDF, European Dermatology Forum; EMSO, European Society of Medical Oncology; EORTC, European Organization for Research and Treatment of Cancer; FESEO, Federation of Spanish Oncology Societies; GEM, Spanish Multidisciplinary Melanoma Group; GS, general surgery; IRCCS, Istituto di Ricovero e Cura a Carattere Scientifico (IRCCS); LR, literature review; MCMCC, Maria Sklodowska-Curie Memorial Cancer Center and Institute of Oncology in Poland; MO, medical oncology; N, no; NCCN, National Comprehensive Cancer Network; NICE, National Institute for Health and Care Excellence; NMWG, New Zealand’s National Melanoma Working Group; P, pathology; PC, primary care; SAKK, Swiss Group for Clinical Cancer Research; SBD, Brazilian Society of Dermatology; SEOM, Spanish Society of Medical Oncology; SFD, French Dermatological Society; SO, surgical oncology; SR, systematic review; RO, radiation oncology; R, radiology, Y, yes.

**Supplementary Table 2.** Recommendations for Self-Skin Examination, Patient Education and Duration of Clinical Skin Surveillance with a Physician.

| **Geographic Region/Guideline** | **Patient Education on Sun Safety** | **Self-Skin Examination** | **AJCC Melanoma Staging Edition** | **Duration of Clinical Skin Surveillance with a HCP** | **Definition of High-Risk Surveillance Group based on AJCC Stage** |
| --- | --- | --- | --- | --- | --- |
| Australia (CCA)^11-17^ | R | R | 8^th^ | 10 years | IIC or higher |
| Brazil (SBD)^18^ | ND | R | 7^th^ | 10 years** | ND |
| Canada (CMC)^19^ | ND | ND | 8^th^ | 5 years | IIB or higher |
| Croatia (CSMO)^20,21^ | R | R | 7^th^ | 10 years | III |
| Europe (EDF/ EADO/EORTC)^22^ | R | R | 8^th^ | Life-long | IIC or higher |
| Europe (ESMO)^23^ | R | R | 8^th^ | NC | IIC or higher |
| France (SFD)^24^ | R | R | 7^th^ | Life-long | III |
| Germany (DDG/DeCOG)^25^ | R | R | 7^th^ | Life-long | IIC or higher |
| Italy (IRCCS Reggio Emilia)^26^ | R | R | 7^th^ | Life-long | IIB or higher |
| New Zealand (NMWG)^27^ | ND | ND | 8^th^ | ND | IIC or higher |
| Poland (MCMCC)^28^ | ND | R | 7^th^ | Life-long | IIB or higher |
| Spain (FESEO)^29^ | R | R | 8^th^ | Life-long | IIB or higher |
| Spain (Network of Catalan and Balearic Melanoma Centers)^30^ | R | R | 7^th^ | 3-10 years (SD) | IIB or higher |
| Spain (SEOM/GEM)^31^ | R | R | 8^th^ | Life-long | IIB or higher |
| Switzerland (SAKK)^32^ | R | R | 7^th^ | 10 years | IIC or higher |
| United Kingdom (BAD)^33^ | R | R | 7^th^ | 1-10 years (SD)* | III |
| United Kingdom (NICE)^34^ | R | R | 7^th^ | 1-5 years (SD)* | IIC or higher |
| United States (AAD)^35^ | ND | R | 8^th^ | Life-long | IIB or higher |
| United States (NCCN)^36^ | R | R | 8^th^ | Life-long | IIB or higher |

AAD, American Academy of Dermatology; AJCC, American Joint Committee on Cancer; BAD, British Association of Dermatologists; CCA, Cancer Council Australia; CMC, Canadian Melanoma Conference; CMSO, Croatian Society for Medical Oncology; DDG/DeCOG, German Dermatological Society and the Dermatologic Cooperative Oncology Group; EADO, European Association of Dermato-Oncology; EDF, European Dermatology Forum; EMSO, European Society of Medical Oncology; EORTC, European Organization for Research and Treatment of Cancer; FESEO, Federation of Spanish Oncology Societies; GEM, Spanish Multidisciplinary Melanoma Group; HCP, healthcare provider; IRCCS, Istituto di Ricovero e Cura a Carattere Scientifico (IRCCS); MCMCC, Maria Sklodowska-Curie Memorial Cancer Center and Institute of Oncology in Poland; NC, no consensus; NCCN, National Comprehensive Cancer Network; ND, not discussed; NICE, National Institute for Health and Care Excellence; NMWG, New Zealand’s National Melanoma Working Group; R, recommended; SAKK, Swiss Group for Clinical Cancer Research; SBD, Brazilian Society of Dermatology; SD, stage dependent; SEOM, Spanish Society of Medical Oncology; SFD, French Dermatological Society.

*Clinical follow-up not required in stage 0. **Recommendation is for patients with stage II melanoma

**Supplementary Table 3.** Recommendations for Number of Clinic Visits Per Year and Physician Specialty to Conduct Melanoma Follow-Up Care

| **Geographic Region and Guideline** | **Recommendations for Frequency of Clinical Follow-Up Visits** | **Recommendations for Physician Specialty to Conduct Follow-Up Care** |
| --- | --- | --- |
| Australia (CCA)^11-17^ | Stage 0: ND  Stage I: q12 mo x 10 yrs  Stage IIA: q6 mo x 2 yrs, then q12 mo x 8 yrs  Stage IIB-IIC: q3-4 mo x 2 yrs, q6 mo x 1 yr, then q12 mo x 5 yrs  Stage III: q3 mo x 2 yrs, q6 mo x 1 yr, then q12 mo x 5 yrs  Stage IV: ND | - Patient should follow-up with a healthcare provider of their choice. - Specialists involved in follow-up clinical care may include general practitioners, dermatologist, surgeons, or medical oncologists. - Providers with experience in skin examination and dermoscopy are preferred. |
| Brazil (SBD)^18^ | Stage 0: NR  Stage I: ND  Stage II: q3-6 mo for 5 yrs, then q12 mo up to 10 yrs | ND |
| Canada (CMC)^19^ | Stage 0-IIA: ND  Stage IIB-IV: q6 mo x 5 yrs | - Medical oncologists, surgical oncologists, dermatologists, and general practitioners with a special interest in melanoma should conduct follow-up surveillance for high-risk (Stage IIB-IV) patients. |
| Croatia (CSMO)^20,21^ | Stage 0: ND  Stage IA: q6 mo x 2 yrs, then q12 mo up to 10 yrs  Stage IB-IIB: q6 mo x 10 yrs  Stage III: q3 mo x 3 yrs, then q6 mo up to 10 yrs  Stage IV: Individualize | - Early-stage melanomas (stage IA) should be under the care of a dermatologist, should not be referred to an oncologist. |
| Europe (EDF/ EADO/ EORTC)^22^ | Stage 0: ND  Stage IA: q6 mo x 3 yrs, then q12 mo for life  Stage IB-IIB: q3-6 mo x3 yrs, then q6 mo up to 10 yrs, then q12 mo for life  Stage IIC-IV (NED): q3 mo x 3 yrs, then q6 mo up to 10 yrs, then q12 mo for life  Stage IV (AM): Individualize or examine q12 weeks | - Follow-up is mainly performed by dermatologists in some European countries - In countries with a limited number of dermatologists, there is insufficient capacity to provide follow-up clinical exams to all melanoma patients. Therefore, general practitioners with additional training in dermoscopy can provide follow-up care to melanoma patients, in collaboration with dermatologists. |
| France (SFD)^24^ | Stage 0: ND  Stage IA-IB: q6 mo x 3 yrs, then q12 mo for life  Stage IIA-IIB: q3-6 mo x 3 yrs, then q12 mo for life  Stage IIC-IIIA: q3 mo x 3 yrs, q6 mo x 2 yrs, then q12 mo for life  Stage IIIB-IIIC: q4 mo x3 yrs, q6 mo in yrs 4-5, then q12 mo for life  Stage IV: ND | ND |
| Germany (DDG & DeCOG)^25^ | Stage 0: ND  Stage IA: q6 mo x 3 yrs, then q12 mo for life  Stage IB-IIB: q3 mo x 3 yrs, then q6 mo in yrs 4-5, then q6-12 mo for yrs 6-10, then q12 mo for life  Stage IIC-IV: q3 mo x 5 yrs, then q6 mo for yrs 6-10, then q12 mo for life | - Follow-up with a dermatologist who is trained in dermoscopy is recommended for clinical skin examinations. |
| Italy (IRCCS Reggio Emilia)^26^ | Stage 0: q12 mo* (continue for life)  Stage IA-IB: q6 mo x 3 yrs, then q12 mo for life  Stage IIA-IIB: q3-6 mo x 3 yrs, then q12 mo for life  Stage IIC-IIA: q3 mo for 3 yrs, q6 mo for 2 yrs, then q12 mo for life  Stage IIIB-IIIC: q4 mo for 3 yrs, q6 mo in yrs 4-5, then q12 mo for life  Stage IV: ND | ND |
| Poland (MCMCC)^28^ | Stage 0: ND  Stage IA: q6–12 mo x 5 yrs, then q12 mo for life  Stage IB-IIC: q3-6 mo x 2-3 yrs, then q6–12 mo x 3 yrs, then q12 mo for life  Stage III: q3 mo x 2 yrs, then q3-6 mo x 3 yrs, then q12 mo for life  Stage IV: Individualize | ND |
| Spain (FESEO)^29^ | Stage 0: q12 mo for life  Stage IA-IIA: q6 mo x 3 yrs, then q12 mo for life  Stage IIB-IIID: q3 mo x 3 yrs, q6 mo for yrs 4-5, then q12 mo for life  Stage IV: Individualize (may depend on tumor board or clinical trial protocol) | - Dermatologists and primary care physicians should conduct outpatient skin surveillance. - The use of dermoscopy in skin surveillance is recommended. |
| Spain (Network of Catalan and Balearic Melanoma Centers)^30^ | Stage 0: q12 mo for 3-5 yrs  Stage IA: q3-6 mo x 2 yrs, then q6 mo x 3 yrs, then q12 mo if needed up to 10 yrs  Stage IB-IIA: q3-6 mo x 3 yrs, then q6 mo for 2 yrs, then q12 mo for 5 yrs  Stage IIB-III: q3-4 mo x 3 yrs, then q6 mo x 2 yrs, then q12 mo for 5 yrs  Stage IV: ND | - Regular dermoscopic monitoring by a dermatologist is recommended |
| Spain (SEOM & GEM)^31^ | Stage 0: ND  Stage IA-IIA: q12 mo for life  Stage IIB-IV: q3-6 mo x 2 yrs, then q3–6 mo x 3 yrs, then q12 mo for life | ND |
| Switzerland (SAKK)^32^ | Stage 0: ND  Stage IA-IB (≤T1N0): q6-12 mo x 3 yrs, then q12 mo until yr 10  Stage IB(T2N0), IIA-IIB: q3 mo x 3 yrs, then q6 mo in yrs 4-5, then q6-12 mo until yr 10  Stage IIC-III: q3 mo x 3 yrs, then q3 mo in yrs 4-5, then q6 mo until yr 10  Stage IV: Individualize | ND |
| United Kingdom (BAD)^33^ | Stage 0: NR  Stage IA: q3-6 mo x 1 yr  Stage IB-IIIA: q3 mo x 3 yrs, then q6 mo x 2 yrs  Stage IIIB-IV (resected): q3 mo x 3 yrs, then q6 mo x 2 yrs, then q12 mo up to 10 yrs  Stage IV (unresectable): Individualize | ND |
| United Kingdom (NICE)^34^ | Stage 0: NR  Stage IA: q3-6 mo x 1 yr  Stage IB-IIB: q3 mo x 3 yrs, then q6 mo x 2 yrs  Stage IIC-III: q3 mo x 3 yrs, then q6 mo x 2 yrs  Stage IV: ND | Use of dermoscopy in follow-up care is recommended. |
| United States (AAD)^35^ | Stage 0: q6-12 mo x 1-2 yrs, then q12 mo for life  Stage IA-IIA: q6-12 mo x 2-5 yrs, then q12 mo for life  Stage IIB-IV: q3-6 mo x 2 yrs, q6 mo x 3 yrs, then q12 mo for life | Dermatologists should collaborate with medical oncologists in advanced melanoma cases. |
| United States (NCCN)^36^ | Stage 0: q12 mo for life  Stage I: q6-12 mo x 5 yrs, then q12 mo for life  Stage IIA: q6-12 mo x 5 yrs, q12 mo for life  Stage IIB-IV: q3-6 mo x 2 yrs, then 3-12 mo x 3 yrs, then q12 mo for life | ND |

AAD, American Academy of Dermatology; AM, active metastases; BAD, British Association of Dermatologists; CCA, Cancer Council Australia; CMC, Canadian Melanoma Conference; CMSO, Croatian Society for Medical Oncology; DDG/DeCOG, German Dermatological Society and the Dermatologic Cooperative Oncology Group; EADO, European Association of Dermato-Oncology; EDF, European Dermatology Forum; EORTC, European Organization for Research and Treatment of Cancer; FESEO, Federation of Spanish Oncology Societies; GEM, Spanish Multidisciplinary Melanoma Group; IRCCS, Istituto di Ricovero e Cura a Carattere Scientifico (IRCCS); MCMCC, Maria Sklodowska-Curie Memorial Cancer Center and Institute of Oncology in Poland; mo, months; NCCN, National Comprehensive Cancer Network; ND, not discussed; NED, no evidence of disease; NICE, National Institute for Health and Care Excellence; NR, not required; q, every; SAKK, Swiss Group for Clinical Cancer Research; SBD, Brazilian Society of Dermatology; SEOM, Spanish Society of Medical Oncology; SFD, French Dermatological Society; yrs, years.

*every 6 months if additional risk factors present (i.e. multiple nevi, personal or family history of melanoma)

**Supplementary Table 4.** Recommendations for Regional Lymph Node Ultrasound, PET-CT, CT-CAP and MRI Imaging Schedules

| **Geographic Region and Guideline** | **Regional Lymph Node Ultrasound** | **PET-CT and/or CT-CAP** | | **MRI Brain** |
| --- | --- | --- | --- | --- |
|  |  | Preferred Modality | Imaging Schedule |  |
| Australia (CCA)^11-17^ | O (in Stage IIC+) | PET-CT | Stage IIC-III: q3-12 mo x 3 yrs | Stage IIC-III: q3-12 mo x 3 yrs |
| Brazil (SBD)^18^ | O (in stage IB+) | NA | NR (only stage 0-I discussed) | ND |
| Canada (CMC)^19^ | Stage IIB-IV: q4-6 mo x 5 yrs | PET-CT^d^ | Stage IIB-IV: q6 mo x 2 yrs, then q12 mo for last 3 yrs | Stage IIB-IV: q12 mo x 5 yrs |
| Croatia (CSMO)^20,21^ | Stage IB to IIB: q6 mo x 5 yrs.  Stage III: q3 mo x 3 yrs, then q6 mo until 5 yrs. | PET-CT^d^ | Stage III: q6 mo x 3 yrs | O |
| Europe (EDF/ EADO/EORTC)^22^ | Stage IB-IIB: q6 mo x 3 yrs  Stage IIC-IV (NED): q3-6 mo x 3 yrs | NP | Stage IIC-IIIC: q6 mo x 3 yrs  Stage IIID: q3-6 mo x 3 yrs  Stage IV (NED): q3 mo x 3 yrs  Stage IV (metastases active): I^f^ | Stage IIC-IIIC: q6 mo x 3 yrs  Stage IIID: q3-6 mo x 3 yrs  Stage IV (NED): q3 mo x 3 yrs  Stage IV (metastases active): I^f^ |
| Europe (ESMO)^23^ | Stage III-IV: If SLN+ | NA | NC | ND |
| France (SFD)^24^ | Stage IIA-III: q3–6 mo x 3 yrs | PET-CT^d^ | Stage IIC-IIIA: q6 mo x 3 yrs (O)  Stage IIIB-IIIC: q6 mo x 3 yrs | ND |
| Germany (DDG/DeCOG)^25^ | Stage IB-IIB: q6 mo x 3 yrs  Stage IIC-IV: q3 mo x 3 yrs | PET-CT | Stage IIC-IV: q6 mo x 3 yrs | Stage IIC-IV: q6 mo x 3 yrs |
| Italy (IRCCS) Reggio Emilia)^26^ | Stage IA: q12 mo x 5 yrs  Stage IB: q6-12 mo x 5 yrs  Stage II: q12 mo x 5 yrs  Stage III: q6 mo x 5 yrs then q12 mo for yrs 5-10 | CT-CAP | Stage II-III: q12 mo x 5 yrs | ND |
| New Zealand (NMWG)^27^ | Stage IB-IIC: q6 mo x 2 yrs, O^a^  Stage III-IV: q6 mo x 2 yrs, O^b^ | CT-CAP^e^ | Stage IIC: q6 mo x 3 yrs. O: q12 mo in yrs 3–5.  Stage IIIA: q6 x 1 yr, then q12 x 2 yrs  Stage IIIB-IV: q3–6 mo x 3 yrs then q12 mo x 2 yrs | Stage IIC: q6 mo x 3 yrs.  Stage IIIA: NR  Stage IIIB-IV: q3-6 mo x 3 yrs, then q12 mo x 2 yrs^g^ |
| Poland (MCMCC)^28^ | O^a^ | CT-CAP | Stage IIB-IV: q6-12 mo x 3 yrs | Stage IIB-III: q12 mo x 3 yrs |
| Spain (FESEO)^29^ | Stage IB-IIID: q3–6 mo x 2 yrs, q6 mo x yrs 3–5^c^  Stage IV: I | CT-CAP | Stage IIB: q6 mo x 3 yrs  Stage IIC-IIID: q3 mo x 1 yr, then q3-6 mo x 2 yrs, then q12 mo x 2 yrs  Stage IV: I^f^ | Stage IIC-IIID: q6 mo x 3 yrs Stage IV: I^f^ |
| Spain (Network of Catalan and Balearic Melanoma Centers)^30^ | ND | CT-CAP | Stage III: q6-12 mo x 3 yrs, then q12 mo x 2 yrs | O |
| Spain (SEOM/GEM)^31^ | Stage IIC-III: q4 mo x 2 yrs, then q6 mo x 3 yrs | CT-CAP | Stage IIB-IV: q3 mo (total duration not specified) | Stage IIB-IV q3 mo (total duration not specified) |
| Switzerland (SAKK)^32^ | Stage I (T2N0), IIA-IIB: q6-12 mo x 5 yrs  Stage IIC-III q6 mo x 5 yrs  Stage IV: I | PET-CT^d^ | Stage IIC-III q6 mo x 5 yrs  Stage IV: I^f^ | Stage IIC-III q6 mo x 5 yrs  Stage IV: I^f^ |
| United Kingdom (BAD)^33^ | ND | ND | ND | ND |
| United Kingdom (NICE)^34^ | ND | ND | Stage IIC-III: Imaging q6 mo x 3 yrs (modality not specified) | ND |
| United States (AAD)^35^ | Stage IB-IIC: O^a^ Stage III-IV: O^b^ | PET-CT^d^ | Stage IIB-IV: q3-12 mo x 3-5 yrs | Stage IIB-IV: q3-12 mo x 3-5 yrs |
| United States (NCCN)^36^ | Stage III: q4 mo x 2 yrs, then q6 mo x 3 yrs^b^ | PET-CT^d^ | Stage IIB-IV: q3-12 mo x 3-5 yrs | Stage IIB-IV: q3-12 mo x 3-5 yrs |

AAD, American Academy of Dermatology; BAD, British Association of Dermatologists; CCA, Cancer Council Australia; CMC, Canadian Melanoma Conference; CMSO, Croatian Society for Medical Oncology; CT-CAP, computerized tomography of the chest, abdomen and pelvis; DDG/DeCOG, German Dermatological Society and the Dermatologic Cooperative Oncology Group; EADO, European Association of Dermato-Oncology; EDF, European Dermatology Forum; EMSO, European Society of Medical Oncology; EORTC, European Organization for Research and Treatment of Cancer; FESEO, Federation of Spanish Oncology Societies; GEM, Spanish Multidisciplinary Melanoma Group; I, individualize imaging schedule based on individual patient factors; IRCCS, Istituto di Ricovero e Cura a Carattere Scientifico (IRCCS); MCMCC, Maria Sklodowska-Curie Memorial Cancer Center and Institute of Oncology in Poland; mo, months; NC, no consensus; NCCN, National Comprehensive Cancer Network; ND, not discussed; NICE, National Institute for Health and Care Excellence; NMWG, New Zealand’s National Melanoma Working Group; NR, not recommended; O, optional/may consider; PET-CT, positron emission tomography and computerized tomography; q, every; R, recommended; SAKK, Swiss Group for Clinical Cancer Research; SBD, Brazilian Society of Dermatology; SEOM, Spanish Society of Medical Oncology; SFD, French Dermatological Society; yrs, years.

^a^ If sentinel lymph node biopsy not performed where clinically indicated

^b^ If sentinel lymph node biopsy was positive but complete lymph node dissection not performed

^c^ Increased US frequency recommended if no SLB performed when indicated or positive SLB without complete lymph node dissection

^d^ PET-CT preferred due to higher sensitivity and specificity, but CT-CAP acceptable if PET-CT not available in geographic region

^e^ CT-CAP preferred due to regional imaging availability, but PET-CT recommended if CT-CAP equivocal or if clinical findings are highly suspicious of metastasis

^f^ Individualize in stage IV metastatic disease based on clinical trial protocol or tumor board recommendations

^g^ Brain MRI or CT head may be used for cranial surveillance

**Supplementary Table 5.** Recommendations for Laboratory Investigations

| **Geographic Region/Guideline** | **Recommendations for Laboratory Investigations** |
| --- | --- |
| Australia (CCA)^11-17^ | NR |
| Brazil (SBD)^18^ | O (S100B in stage IB+) |
| Canada (CMC)^19^ | NR |
| Croatia (CSMO)^20,21^ | Stage IB-IIB: S100B q6 mo x 5 yrs.  Stage III: S100B q3 mo x 3 yrs, then q6 mo up to 5 yrs. |
| Europe (EDF/EADO/EORTC)^22^ | Stage IB-IV (NED): LDH and S-100 q3-6 mo x 3 yrs |
| Europe (ESMO)^23^ | O (may consider S100B) |
| France (SFD)^24^ | NR |
| Germany (DDG/DeCOG)^25^ | Stage IB-IIB: S100B q3 mo x3 yrs  Stage IIC-IV: S100B q3 mo x3 yrs, then q6 mo for yrs 4-5 |
| Italy (IRCCS Reggio Emilia)^26^ | NR |
| New Zealand (NMWG)^27^ | ND |
| Poland (MCMCC)^28^ | O (CBC, LFTs and LDH) |
| Spain (FESEO)^29^ | Stage IB-IIA: LDH q6 mo x 3 yrs, q12 mo afterwards  Stage IIB-IIID: LDH q3 mo x 3 yrs, q6 mo for yrs 4-5, q12 mo afterwards  Stage IV: LDH q3 mo |
| Spain (Network of Catalan and Balearic Melanoma Centers)^30^ | Stage IA: LDH q6-12 mo x 2 yrs, then q12 mo x 2 yrs.  Stage IB-IIA: LDH q3-6 mo x 3 yrs, then q6 mo x 2 yrs, then q12 mo x 5 yrs.  Stage IIB-III: LDH q3-6 mo x 3 yrs, then q6 mo x 2 yrs, then q12 mo x 5 yrs. |
| Spain (SEOM/GEM)^31^ | O |
| Switzerland (SAKK)^32^ | Stage I (T2N0), IIA-IIB: S100B q6-12 mo x 5 yrs  Stage IIC-III S100B q6 mo x 5 yrs  Stage IV: S100B (I) |
| United Kingdom (BAD)^33^ | ND |
| United Kingdom (NICE)^34^ | ND |
| United States (AAD)^35^ | NR |
| United States (NCCN)^36^ | NR |

AAD, American Academy of Dermatology; BAD, British Association of Dermatologists; CCA, Cancer Council Australia; CMC, Canadian Melanoma Conference; CMSO, Croatian Society for Medical Oncology; DDG/DeCOG, German Dermatological Society and the Dermatologic Cooperative Oncology Group; EADO, European Association of Dermato-Oncology; EDF, European Dermatology Forum; EMSO, European Society of Medical Oncology; EORTC, European Organization for Research and Treatment of Cancer; FESEO, Federation of Spanish Oncology Societies; GEM, Spanish Multidisciplinary Melanoma Group; IRCCS, Istituto di Ricovero e Cura a Carattere Scientifico (IRCCS); MCMCC, Maria Sklodowska-Curie Memorial Cancer Center and Institute of Oncology in Poland; mo, months; NCCN, National Comprehensive Cancer Network; ND, not discussed; NICE, National Institute for Health and Care Excellence; NMWG, New Zealand’s National Melanoma Working Group; NR, not recommended; O, optional; q, every; SAKK, Swiss Group for Clinical Cancer Research; SBD, Brazilian Society of Dermatology; SEOM, Spanish Society of Medical Oncology; SFD, French Dermatological Society; y, years.

**Supplementary Figure 1.** Preferred Reporting Items for Systematic Reviews and Meta-Analyses flowchart depicting the MEDLINE and Embase database search, title and abstract screening, full text review, manual citation search, studies excluded and included studies.


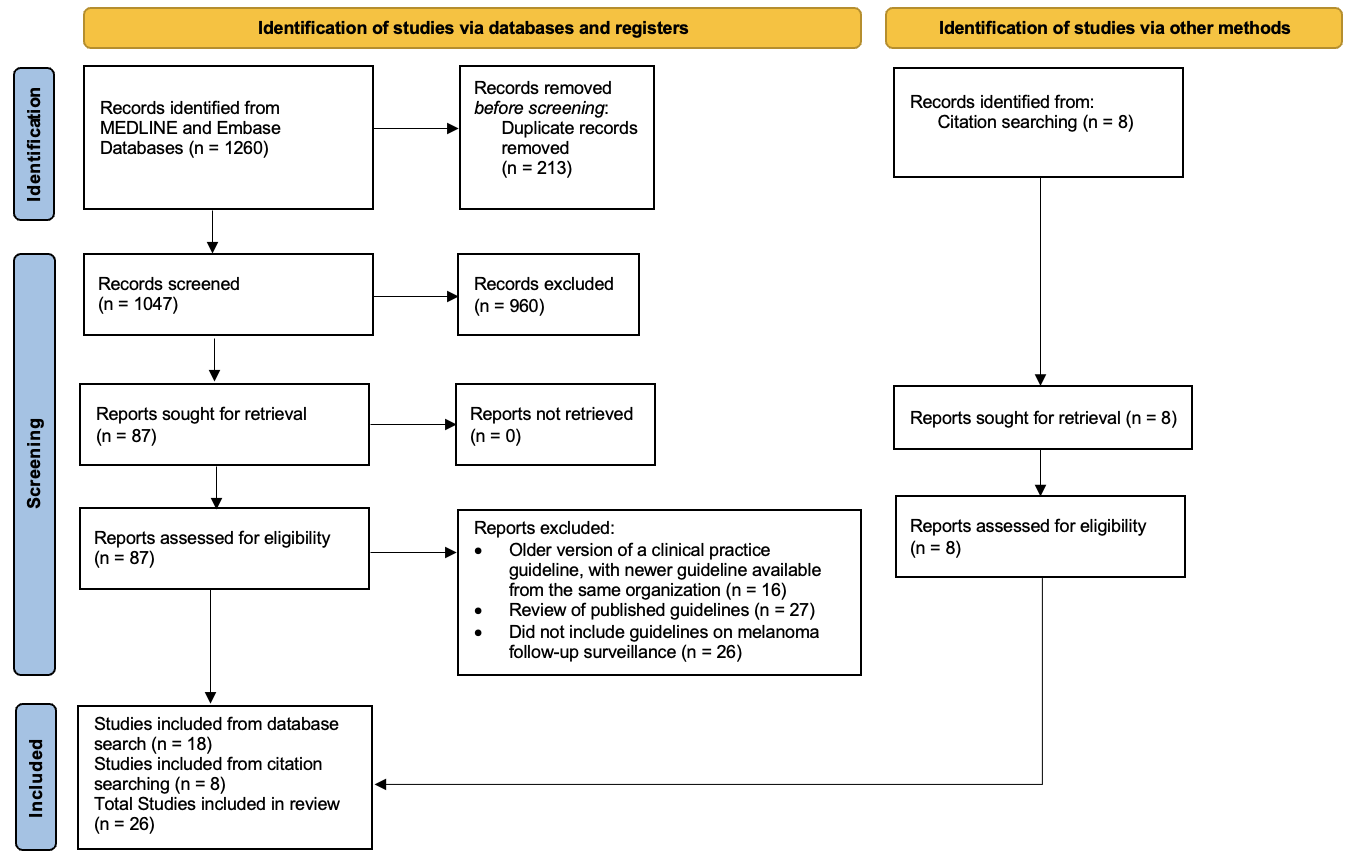

Supplement: Supplementary Material 1 - Supplemental material for Surveillance After a Previous Cutaneous Melanoma Diagnosis: A Scoping Review of Melanoma Follow-Up Guidelines [file sj-docx-1-cms-10.1177_12034754231188434.docx]
