# Supplementary figures and images for: Surveillance After a Previous Cutaneous Melanoma Diagnosis: A Scoping Review of Melanoma Follow-Up Guidelines
Source: J Cutan Med Surg. 2023 Jul 25;27(5):516–25. doi: 10.1177/12034754231188434 (PMC10617001; doi:10.1177/12034754231188434)

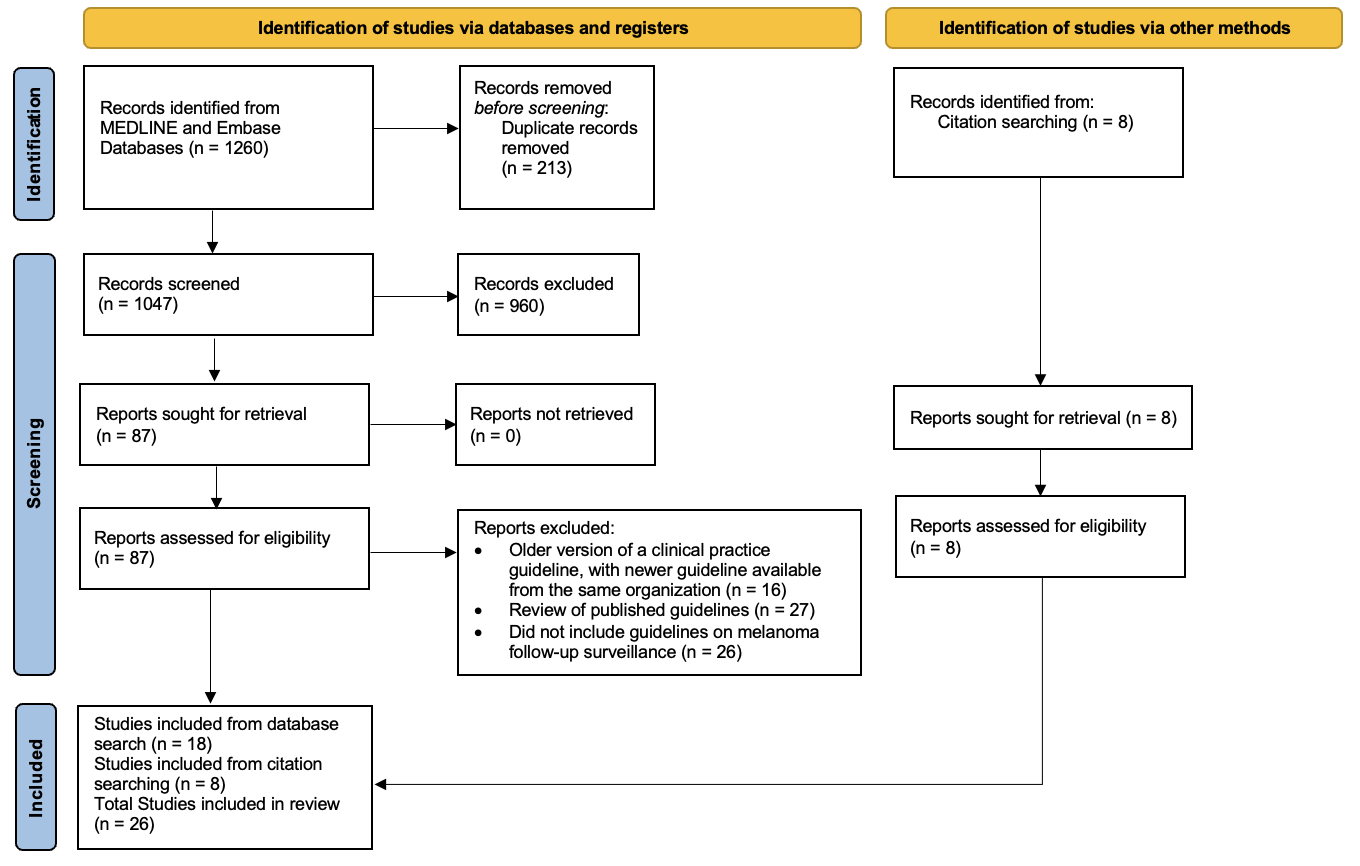

Supplement: Figure S1 - Supplemental material for Surveillance After a Previous Cutaneous Melanoma Diagnosis: A Scoping Review of Melanoma Follow-Up Guidelines [file sj-png-1-cms-10.1177_12034754231188434.png]
